# Overexpression of Multifunctional Protein p32 Promotes a Malignant Phenotype in Colorectal Cancer Cells

**DOI:** 10.3389/fonc.2021.642940

**Published:** 2021-05-31

**Authors:** Carlos Alejandro Egusquiza-Alvarez, M. Cristina Castañeda-Patlán, Sara Albarran-Gutierrez, Héctor Gonzalez-Aguilar, Angela P. Moreno-Londoño, Vilma Maldonado, Jorge Melendez-Zajgla, Martha Robles-Flores

**Affiliations:** ^1^ Departamento de Bioquímica, Facultad de Medicina, Universidad Nacional Autónoma de México (UNAM), Mexico City, Mexico; ^2^ Epigenetics and Functional Genomics Laboratories, National Institute of Genomic Medicine, Mexico City, Mexico

**Keywords:** p32/gC1qR/HABP1, colorectal cancer, migration, cell death, clonogenesis, tumorigenesis, Akt/mTOR pathway

## Abstract

p32 is a multifunctional and multicompartmental protein that has been found upregulated in numerous adenocarcinomas, including colorectal malignancy. High levels of p32 expression have been correlated with poor prognosis in colorectal cancer. However, the functions performed by p32 in colorectal cancer have not been characterized. Here we show that p32 is overexpressed in colorectal cancer cell lines compared to non-malignant colon cells. Colon cancer cells also display higher nuclear levels of p32 than nuclear levels found in non-malignant cells. Moreover, we demonstrate that p32 regulates the expression levels of genes tightly related to malignant phenotypes such as *HAS-2* and *PDCD4*. Remarkably, we demonstrate that knockdown of p32 negatively affects Akt/mTOR signaling activation, inhibits the migration ability of colon malignant cells, and sensitizes them to cell death induced by oxidative stress and chemotherapeutic agents, but not to cell death induced by nutritional stress. In addition, knockdown of p32 significantly decreased clonogenic capacity and *in vivo* tumorigenesis in a xenograft mice model. Altogether, our results demonstrate that p32 is an important promoter of malignant phenotype in colorectal cancer cells, suggesting that it could be used as a therapeutic target in colorectal cancer treatment.

## Introduction

Colorectal Cancer (CRC) is the third most frequently diagnosed type of malignancy and the second leading cause of cancer death worldwide, according to estimates from International Agency for Research on Cancer in 2018 (1). Surgery and chemotherapy are the first choices of treatment for patients with this disease. However, the prognosis for CRC is unfavorable in many cases, particularly for patients in more advanced stages. Therefore, finding new therapeutic strategies is essential. Targeted therapy is a new option that has successfully prolonged the survival of CRC patients. These therapies aim to inhibit the proliferation, differentiation, and migration of tumor cells and regulate the tumor microenvironment by acting on specific molecular targets. Identifying the multiple and heterogeneous molecular mechanisms that promote malignant phenotypes will help interfere more efficiently, oncogenic cellular processes improving personalized and targeted cancer therapy (2).

p32, also denominated “Receptor of the globular head of C1q (gC1qR)” (3) and “Hialuronic Acid Binding Protein-1” (HABP1) (4), is an acidic (3, 5) and highly conserved (6–9) protein that has been found over-expressed in various cancer types (10). The protein is synthesized as a 282 aminoacid residue pre-protein. After synthesis, it undergoes a post-translational maturation process by proteolytic cleavage of 73 residues at its N-terminus, remaining as a mature protein with 209 aminoacid residues and an apparent molecular weight in SDS-PAGE of 32 kDa (11). From crystallization and 3D protein structure determination, it could be known that p32 forms a doughnut-shaped homotrimer, with an asymmetric charge distribution on its surface (7). The protein has a mitochondrial localization signal at its N-terminus (12). However, multiple studies have shown that depending on the cell context, the mature variant is not only found in the mitochondrial matrix but can also be found in the nucleus (13, 14) as well as on the cell surface (15–18). Due to this, p32 is considered as a multicompartmental protein (15). In addition, one of the most important features of p32 is the ability to bind multiple unrelated ligands at each of the subcellular localization where it is found. Among the most important ligands are included: serum proteins as C1q, H-kininogen (HK) and fibrinogen; extracellular matrix components as hyaluronic acid; multiple viral and bacterial proteins; mitochondrial proteins as short mitochondrial ARF (smARF), cytochrome B2 and HRK; cytosol proteins as PKC and nuclear proteins as pre-mRNA splicing factor (SF2/ASF), Lamin B receptor and p53 (14, 19, 20). Precisely because it is a multicompartmental, multiligand, and highly flexible protein (21), it has been assigned many diverse functions in each of its subcellular locations. Within the main functions of p32 are the maintenance of oxidative phosphorylation (OXPHOS) (22), regulation of cell death or survival through modulating mitochondrial dynamic (20), splicing regulation (23) as well as participation in several processes related to inflammation, infection, and immune response (19).

Interestingly, p32 has been found upregulated in several types of malignancies, including thyroid, pancreas, stomach, esophagus, breast, ovary, testis, skin, lung and colorectal human adenocarcinomas relative to non-malignant counterparts. These findings suggest an important role for p32 in cancer pathology (18, 24). In fact, several studies have demonstrated that p32 is a promoter of malignant phenotypes in breast (25), skin (26), lung (27), hepatic (28), and pancreas (29) cancer cells. A recent Systematic Multiomics Analysis showed significantly lower colorectal cancer patient survival in the high p32 expression group compared to the low p32 expression group, suggesting a role for the protein as a promoter of malignancy in this type of tumor (30). However, the functions of p32 in colorectal cancer cells have not been reported so far.

Here we show that p32 is overexpressed in colorectal cancer cells compared to non-malignant colon cells. Our findings demonstrate that high expression levels of p32 protein promotes malignant phenotype through regulating the expression of many key cancer related genes and by enhancing mTORC activation, migration, resistance to cell death, and promoting clonogenic and tumorigenic capacity of colorectal cancer cells. These results strongly suggest that p32 is a potential target for the therapy of CRC.

## Materials and Methods

### Antibodies and Reagents

The antibodies used in this study include the following: rabbit anti-phosphor-mTOR, rabbit anti-mTOR, mouse anti-Phospho-p70 S6 Kinase, rabbit anti-p70 S6 kinase, rabbit anti-CD44, rabbit anti-cleaved PARP and rabbit anti-cleaved caspase-3 were all obtained from Cell Signaling Technology (Danvers, MA, USA). Mouse-anti-p32 (gC1qR) antibody was obtained from Abcam (Cambridge, MA, USA). Rabbit anti-Akt and mouse anti-Phospho-Akt antibodies were purchased from Santa Cruz Biotechnology (Dallas, TX, USA). Goat anti-mouse and anti-rabbit IgG-horseradish peroxidase-conjugates were from Pierce (Rockford, IL, USA). Mouse monoclonal anti-actin was a gift from Dr. José Manuel Hernandez-Hernández (CINVESTAV-IPN). Temsirolimus (CCI-779) and 5-fluorouracil (5-FU) were purchased from Sigma-Aldrich (St. Louis, MO, USA).

### Cell Culture

The colon cancer cell lines used in this work were the following: the human RKO malignant cells exhibit normal canonical Wnt signaling (expressing wild-type APC protein) and are the prototype of BRAF-driven colon cancer cells (Braf V600E and PIK3CA H1047R mutations) (31). Human carcinoma SW480 cells express a truncated version of APC, has constitutively active canonical Wnt signaling and is the prototype of KRAS-driven colon cancer cells (KRAS G12V, APC A1457T/K1462R, FGFRS400R, TP53 R273H, and STK11 G58S mutations) (31). 112CoN non-malignant and malignant RKO cells were cultured in Dulbecco’s modified Eagle’s medium (DMEM) supplemented with 10% Fetal Bovine Serum (FBS), antibiotics (200 mg/ml Streptomycin and 120 mg/ml Penicillin), and 2mM L-glutamine. SW480 and SW620 cells were maintained in DMEM F-12 supplemented with 5% FBS, antibiotics, and 2mM glutamine. All cells were obtained from American Type Culture Collection (ATCC) (Manassas, VA, USA). They were authenticated in June 2017 by Short Tandem Repeat DNA profiling analysis performed at the Instituto Nacional de Medicina Genomica (INMEGEN), Mexico City.

### Knockdown of p32

Knockdown of p32 in RKO and SW480 cells was obtained by stable transfection of the *RNAi-Ready pSIREN-RetroQ* plasmid from Clontech, which generates the shRNA for p32 protein. As a control, RKO and SW480 cells, stably transfected with the same plasmid but without the shRNA (empty plasmid) sequence, were used.

### Western Blot

Cells were lysed with RIPA lysis buffer (50 mM Tris-HCl pH=7.4, 150 mM NaCl, 0.1% SDS, 1% NP-40, 0.25% Na-deoxycholate, 1 mM EDTA) supplemented with protease and phosphatase inhibitors for 15 min at 4°C. After centrifugation (13 000 rpm) for 15 min at 4°C, 40 μg of the whole-cell lysate (supernatant) were separated by 8, 10 or 15% SDS-polyacrylamide gel electrophoresis (SDS-PAGE) followed by electrophoretic transfer to nitrocellulose membranes (Bio-Rad). The membranes were blocked with 5% non-fat dry milk or 3% BSA in TBS and incubated overnight at 4°C with the corresponding primary antibody. Detection was achieved using the SuperSignal Kit (Pierce) with a horseradish peroxidase-conjugated second antibody. Actin was used as a control for equal loading.

### RT-PCR

RNA extraction was carried out by using Trizol reagent (Invitrogen). RT-PCR was carried out with SuperScript™ III RT/Platinum^®^ Taq kit, according to the manufacturer’s instructions. GAPDH was reverse transcribed under the same conditions to be used as control. The primers used for RT-PCR are listed in **Table 1**.

**Table 1:** List of the primers used in RT-PCR.

Gene name	Foward primers 5’-3’	Reverse primers 5’-3’
*C1QBP* (p32)	GCCGGGGAAAAAATCACGGTC	CACTCTCAGCCTCGTCTTCTTGTC
*HAS-2*	CAGCCTCATCTGTGGAGATGGT	TCCCAGAGGTCCACTAATGCAC
*TNSF-15*	CTCTGCACTGGGAACATGAACT	TTGGCTCAGGGTAGCTGTCTGT
*HOOK-1*	CCTGGTACCGAGCTTTCCTG	TGTCTGCAGCCAGATCATGAGG
*GAPDH*	CATCTCTGCCCCCTCTGCTGA	GGATGACCTTGCCCACAGCCT

### Confocal Immunofluorescence Microscopy

Cells were fixed with 1% paraformaldehyde for 10 minutes and then permeabilized and blocked simultaneously with PBS- 0.3% Triton X-100- 3% BSA for 1 hour. Next, incubation with primary antibody was performed overnight at 4°C and then with the secondary antibody for 2 h at room temperature. Immunofluorescence images were captured by confocal microscopy (Nikon A1R+ STORM). The image processing and analysis were done by using the programs NIS Elements Viewer and Image J (version 1.47b obtained from the National Institutes of Health website (http://imagej.nih.gov/ij/).

### Flow Cytometry Analysis

Cells were detached and dissociated in 10 mM EDTA solution and subsequently centrifuged and resuspended in FACS buffer (4% Fetal Bovine Serum in PBS). The cells (2 X 10^5^ cells for each condition) were permeabilized with Triton X-100. The primary anti-p32 mouse antibody (1:50, Chemicon) was added and incubated for 30 minutes at 4°C. Next, two washes with cold FACS buffer were performed. Subsequently, the secondary anti-mouse-FITC antibody (1: 100) was added and incubated for 30 minutes at 4°C (protected from light). Cells were washed three times and fixed with 4% paraformaldehyde (PFA). The cells stained with the secondary antibody alone were used as a negative control. Cells were acquired in an Attune Nxt (Thermo Scientific, Waltham, MA, USA), and data were analyzed with the software FlowJo (Tree Star^®^, Ashland, OR, USA).

### Gene Microarray

Total RNA samples from the RKO and SW480 cell lines, both control and p32-silenced cells, were sent to the INMEGEN (National Institute of Genomic Medicine) for analysis by gene microarrays. RNAs with RNA Integrity values (RIN) higher than 9 were analyzed using the Affymetrix platform Gene ST 1.0, as recommended by the manufacturer. The primary analysis was performed using the AltAnalyze platform for normalization, summarization, and differential expression analysis (32). The results were subsequently analyzed using the following bioinformatic programs: Ingenuity Pathway Analysis (IPA), The Database for Annotation, Visualization and Integration Discovery (DAVID), Network Analyst, The Candidate Cancer Gene Database (CCGD), and the bioinformatic tool UALCAN of The Cancer Genome Atlas Program (TCGA).

### Wound Healing Assay

Briefly, RKO or SW480 sh-control or sh-p32 cells were seeded to confluence on 24-well culture plates for 48 h. Next, a scratch was produced using a sterile pipette tip. The cells were then washed with PBS and incubated with serum-free medium at 37°C for 24 hours. Photographs were taken at 0 and 24 hours after scraping the cell monolayer.

### Viability Assay and Apoptosis Analysis

MTT assay was used for the viability evaluation. 1.8 x 10^4^ RKO sh-control or RKO sh-p32 cells/well were seeded on a 96-well plate and grown for 24 h. In the case of SW480 cell line, 2 x 10^4^ sh-control or sh-p32 cells were seeded on a 96-well plate for 48 h. RKO cells were then treated with H_2_O_2_ at concentrations of 8, 4, 2, 1, 0.5, 0.25, 0.125, 0 mM for 12 h to assess the effect of oxidative stress. On the other hand, SW480 cells were treated with H_2_O_2_ at concentrations of 8, 4, 2, 1, 0.5, 0 mM for 16 h to assess the effect of oxidative stress too. To evaluate the effect of chemotherapeutic agents in viability, the cells were treated with CCI-779 (Temsirolimus) at concentrations of 0, 5, 10, 16, 20, 25, 35 μM for RKO cells or 0, 5, 10, 12.5, 16, 20, 25, 35 for SW480 cells for 24 h. In addition, RKO cells were treated with 5-fluorouracil (5-FU) at concentrations of 0, 0.2, 1, 5, 10, 50, 100, 500, 1000, 2500, 5000 μM and SW480 cells were treated with 5-FU at concentrations of 0, 0.2, 1, 5, 10, 50, 100, 500, 1000, 2500 μM for 72 h. In starving experiments, the cells were treated with serum-free medium or with Hanks’ balanced salt solution (HBSS) for 12 h for RKO cells or 48 hours for SW480 cells to induce nutritional stress. After treatments, 0.5 mg/ml MTT was added to the cells for 3 h at 37°C, protected from light. MTT-formazan crystals were dissolved in acid isopropanol pH=4 and quantified by spectrophotometry at 570 nm. In addition, apoptotic cell death was examined by Western blotting to detect the presence of cleaved caspase-3 and/or cleaved PARP protein.

### Evaluation of the Akt/mTOR Cell Signaling Pathway

3 x 10^5^ RKO sh-control or sh-p32 cells were seeded on a 6-well plate and grown for 48 hours. After that time, the cells were starved for 16 hours with serum free media. The cells were then stimulated with 10% FBS-DMEM for 1, 3 or 6 h to evaluate the activation of Akt/mTOR/p70 S6 Kinase cell signaling pathway by Western Blotting. For evaluation of the rate of Akt, mTOR and p70 S6 Kinase activation at longer times, 1 x 10^6^ RKO or SW480 (sh-control or sh-p32) were seeded in p60 plates and grown in 10% FBS-media for 48 hours. The cells were lysed and the levels of phosphorylated Akt, mTOR and p70-S6 Kinase protein were evaluated by Western blotting.

### Colony Formation Assay

1 x 10^2^ RKO sh-control, RKO sh-p32, SW480 sh-control or SW480 sh-p32 cells per well were seeded in 96-well-plate in DMEM. The cells were cultured at 37°C for 10 days. After that time, the colonies obtained were fixed with absolute methanol and stained with a 0.5% violet crystal- 25% methanol solution. The number of colonies was quantified under the light microscope.

### Tumorigenesis in Nude Mice

Six-week-old male nude mice were s.c. injected in their left flank with 1x 10^6^ RKO or SW480 sh-control cells, and in their right flank with 1 x 10^6^ RKO or SW480 sh-p32 cells. After 4 weeks, euthanasia was carried out. The tumors were removed from the animals, and tumoral weight was determined. The experiment was performed under the guidelines for the use and care of laboratory animals.

### Statistical Analysis

All experiments were repeated at least three times using different cell preparations. The data are expressed as the mean ± standard error of the mean (SEM). Statistical data analysis was performed using Student’s t-test or a one-way ANOVA with Bonferroni’s multiple comparison test. All statistical analyses were performed using GraphPad Prism 6. A value of p < 0.05 was considered statistically significant.

## Results

### p32 Protein Is Over-Expressed in Colon Cancer Cells Compared to Non-Malignant Colon Cells

**Figure 1:**
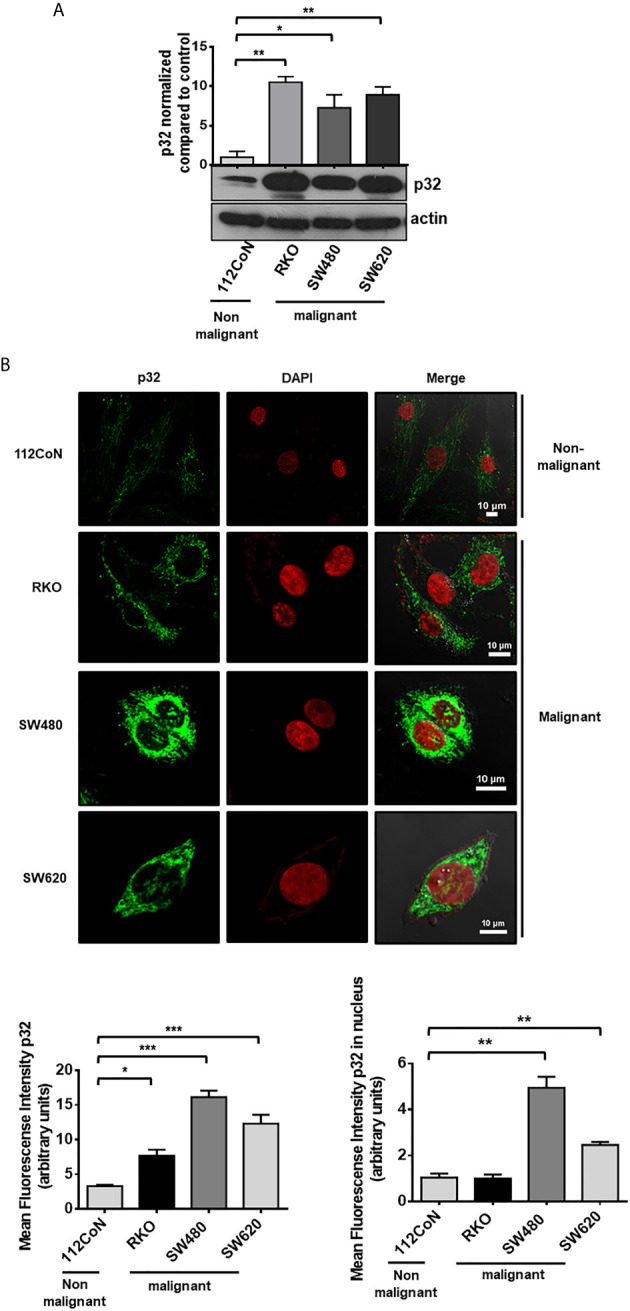
p32 protein is overexpressed in malignant colon cells relative to non-malignant cells. **(A)** The expression of p32 was evaluated in colon non-malignant 112CoN and colon malignant RKO, SW480, and SW620 cells by Western blot. Actin was used as a control for equal loading. Densitometric analysis was performed to quantify the change in p32 expression levels. The data is presented as the mean values ± SEM from at least three independent experiments, *p < 0.05, **p < 0.01. **(B)** 112CoN, RKO, SW480, and SW620 cells were fixed with 1% PFA and permeabilized with 0.3% Triton X-100. The intracellular expression of p32 was examined by immunofluorescence confocal microscopy. p32 nuclear expression was assessed by merge analysis between the p32 and DAPI signals. Scale bar, 10 μm. Representative images from 3 independent experiments are shown. The quantification of p32 expression levels in 112CoN, RKO, SW480, and SW620 cells, measured by mean fluorescence intensity in arbitrary units from analysis of immunofluorescence confocal microscopy images, is also shown. The data is presented as the mean values ± SEM from at least three independent experiments, *p < 0.05, **p < 0.01, ***p < 0.001.

To investigate the role of p32 in colon cancer cells, we first compared the expression level of the protein between malignant and non-malignant colon cell lines by Western blotting (WB). This analysis revealed that colon cancer cell lines (RKO, SW480, and SW620) show higher expression of p32 than non-malignant colon cell line (112CoN) (**Figure 1A**). A similar result was obtained using confocal immunofluorescence microscopy (**Figure 1B**), displaying p32 a punctate intracellular distribution, probably confined to an organelle within cancer cells, while its distribution appears to be coupled to fibers in 112CoN cells. Interestingly, a greater nuclear localization of the protein was found in malignant cells than in non-malignant cells (**Figure 1B**).

### Knockdown of p32 Induces Changes in the Expression Levels of Proteins Related to the Malignant Phenotype in RKO and SW480 Cells

**Figure 2:**
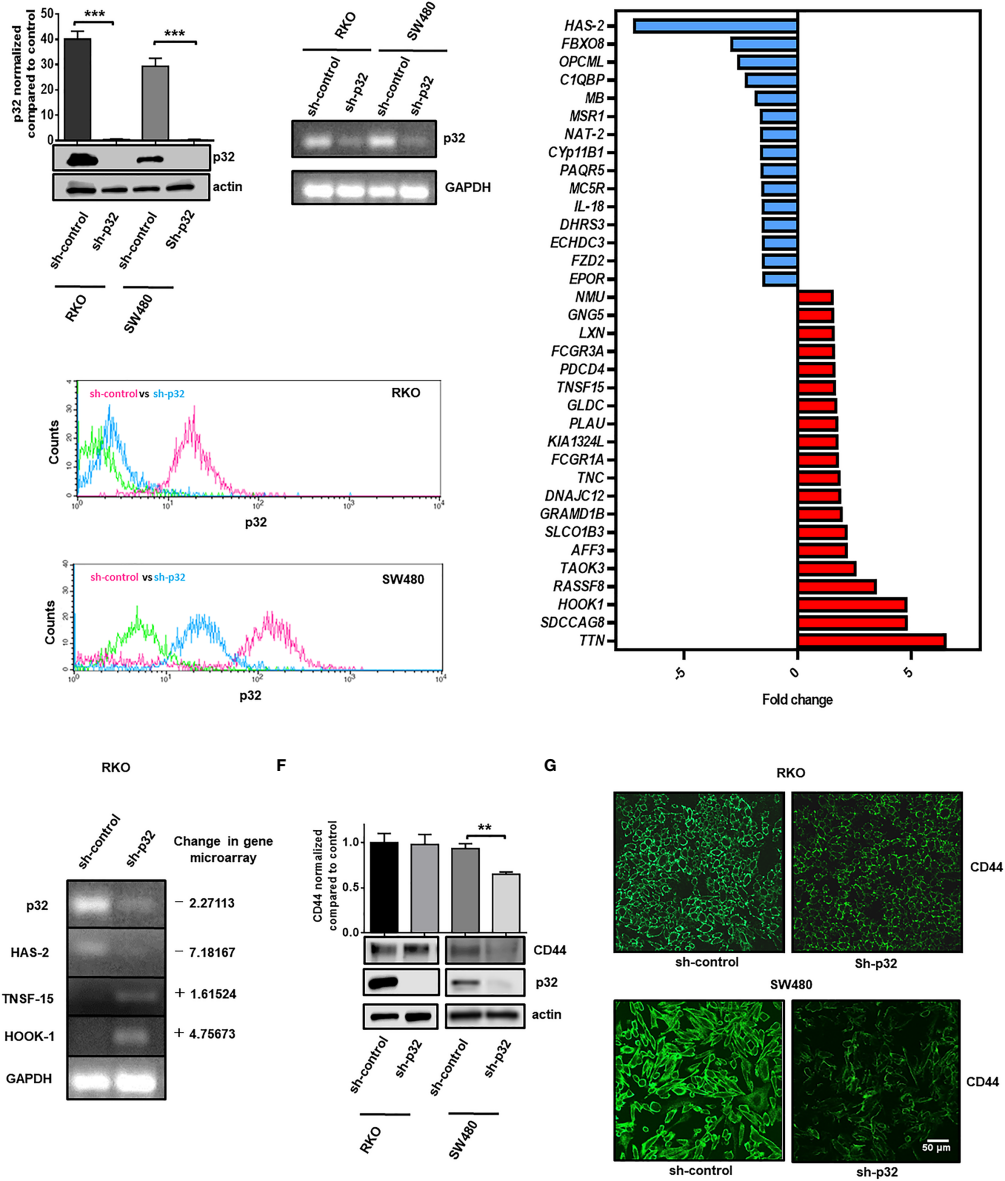
Knockdown of p32 induces changes in the expression levels of proteins related to the malignant phenotype in RKO and SW480 cells. **(A)** The efficiency of p32 knockdown was evaluated in RKO and SW480 cells by Western blot. Actin was used as a control for equal loading. Densitometric analysis was performed to quantify the change in p32 expression levels. The data is presented as the mean values ± SEM from at least three independent experiments, ***p < 0.001. Knockdown efficiency was also examined by RT-PCR **(B)** and flow cytometry **(C)**. **(D)** Fold change of expression graphic of genes related to the malignant phenotype that were downregulated (blue) or upregulated (red) in RKO sh-p32 cells with respect to RKO sh-control. The fold change values were determined from the gene microarray analysis developed as described in the “material and methods” section. **(E)** Changes in the levels of primary transcripts of *C1QBP* (p32), *HAS-2*, *TNSF-15*, and *HOOK-1* were evaluated by RT-PCR from total RNA extracted from RKO sh-control and RKO sh-p32 cells. **(F)** The expression level of CD44 was evaluated in RKO and SW480 sh-control and sh-p32 cells by Western blot. p32 levels were also evaluated for each condition to verify knockdown efficiency. Actin was used as a control for equal loading. Densitometric analysis was performed to quantify the change in CD44 expression levels. The data is presented as the mean values ± SEM from at least three independent experiments, **p < 0.01. **(G)** CD44 expression in RKO and SW480 control and knockdown of p32 cells was also evaluated by immunofluorescence microscopy. Representative images from 3 independent experiments are shown. Scale-bar, 50 μm.

Because of the high expression of p32 found in colon cancer cells, we investigated if the protein could have any effect in promoting the malignant phenotype in colon cancer cells. To evaluate this, we decided to knock down the protein in the RKO and SW480 cell lines through stable transfection of a plasmid that encodes a shRNA against p32. Knockdown efficiency was evaluated using WB. This technique confirmed that expression of p32 was considerably decreased in the RKO and SW480 cells transfected with the shRNA p32 with respect to those transfected with the empty plasmid (**Figure 2A**). Results were further verified by RT-PCR (**Figure 2B**) and flow cytometry with cell permeabilization (**Figure 2C**).

In order to find out whether blocking the expression of p32 would also affect the expression of other genes, a gene microarray analysis was performed. The results obtained showed that 94 sequences changed their expression in RKO sh-p32 with respect to RKO sh-control (**Supplementary Table 1**). The transcription level of 53 genes was upregulated, while 41 genes were downregulated. Through bioinformatic analysis using the programs described in the *Materials and Methods* section, we found that the modified genes are associated with lipid metabolism, small molecule biochemistry, and gene expression. We found that 35 of the modified genes are closely related to malignant phenotypes (**Figure 2D**). Interestingly, it was also found that p32 depletion affected the levels of some genes reported with tumor suppressor function such as *TNSF-15*, *HOOK-1*, and *PDCD4*, whose expression increased, while the levels of other genes involved in promoting the malignant phenotype such as *HAS-2* and *MSR-1* were decreased (**Table 2**). To corroborate the results of the gene microarray, we evaluated by RT-PCR the change in the levels of the primary transcript of two genes that were decreased by knockdown of p32 (*C1QBP* (p32) and *HAS-2*) and of two genes that were found increased (*TNSF-15* and *HOOK-1*). The results obtained from RT-PCR were consistent with the results previously obtained in the gene microarray, as shown in **Figure 2E**.

**Table 2:** Genes associated with the malignant phenotype that changed their expression in RKO knock down p32 cells.

genes	Change in kdp32
*HAS-2*	**- 7.182**
*MSR1*	**- 1.607**
*EPOR*	**- 1.501**
*PDCD4*	**+ 1.592**
*GLCD*	**+ 1.669**
*TNSF-15*	**+ 1.615**
*HOOK-1*	**+ 4.756**

CD44 is a protein closely related to p32, whose fundamental ligand is hyaluronic acid. Hyaluronic acid binding to CD44 has been shown to increase expression levels of CD44 protein (28, 33). Interestingly, we found that, as a consequence of the knockdown of p32, the expression levels of HAS-2 (hyaluronic acid synthetase-2) were decreased (**Supplementary Table 1**). Taking into account these considerations, we asked whether CD44 expression would be affected in p32 knockdown cells. To investigate this, we evaluate CD44 levels by WB in RKO and SW480 control and p32 -depleted cells. Remarkably, we found that CD44 levels were decreased in SW480 p32-silenced cells compared to control cells. However, no significant difference was found in the RKO cell line (**Figure 2F**). These results were confirmed by immunofluorescence microscopy (**Figure 2G**). Taken together, our data demonstrate that p32 can regulate the expression level of different genes associated with the malignant phenotype in colon cancer cells.

### Knockdown of p32 Negatively Affects the Activation of the Akt/mTOR Signaling Pathway

It has been reported in pancreatic and lung cancer cells that stimulation with growth factors induces the translocation of p32 to membrane lipid rafts where its interaction with CD44 favors the autophosphorylation and activation of Receptor Tyrosine Kinases (RTKs) (27, 29). It has also been established that lipid raft proteins may play important roles in cell signaling and cell–cell interaction, and interestingly, Arielly SS. et al. (34) have reported that p32 is the most abundant protein present in caveolin lipid rafts of colon cancer cells. The Phosphatidyl-inositol-3 kinase (PI3K)/Akt or the Ras/ERK signaling pathways are activated downstream by growth factors and converge in mTORC activation to promote cell survival, proliferation and growth. The human RKO and primary SW480 cell lines used in our study, have oncogenic driver mutations that results in mTORC1 activation (31). In view of this, we investigated whether p32 silencing affects the activation of mTOR. We made use of several antibodies to detect by Western blotting the activation status of Akt (phosphorylated at Ser 473), mTOR (at Ser2448) and the typical substrate of mTORC1, the p70 ribosomal S6 protein Kinase (p70-S6K, at Thr389) (35).

**Figure 3:**
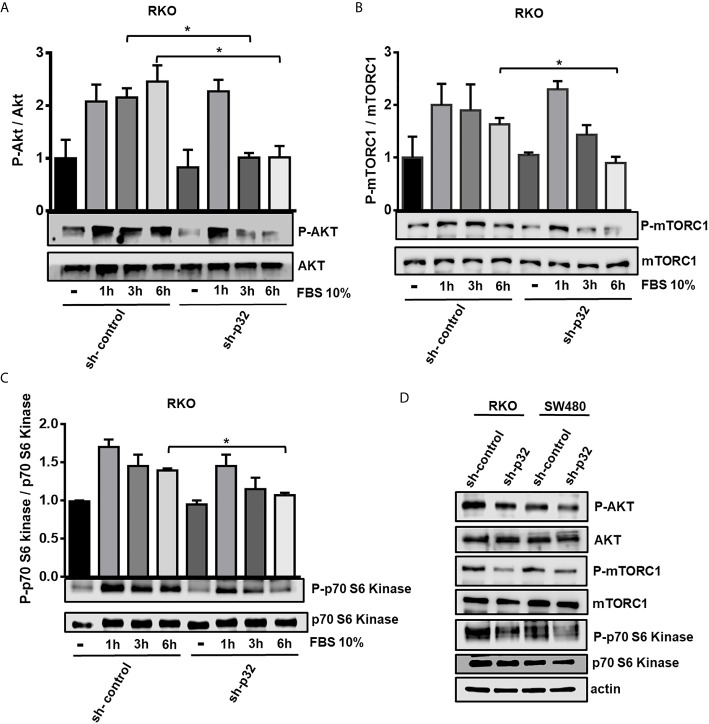
Knockdown of p32 negatively affects the activation of the Akt-mTor signaling pathway in RKO and SW480 cells. 3x10^5^ RKO sh-control or sh-p32 cells were seeded on a 6-well plate and grown for 48 hours. After that time, the cells were starved for 16 hours with serum free media. Next, cells were stimulated with 10% FBS-DMEM for 1, 3 or 6 hours to evaluate the activation of Akt **(A)** mTOR **(B)** and p70-S6 Kinase **(C)** proteins by Western blot. Densitometric analysis was performed to quantify the levels of both phosphorylated and total proteins. The levels of each phosphorylated protein were normalized with respect to its corresponding total protein. The data is presented as the mean values ± SEM from three independent experiments, *p < 0.05. **(D)** For evaluation of the rate of activation of the AKT-mTOR-p70S6 Kinase signaling pathway at longer times, 1x10^6^ RKO or SW480 (sh-control or sh-p32) were seeded in p60 plates and grown in 10% FBS-media for 48 hours. After that time, the cells were lysed and the levels of activation of AKT, mTOR and p70-S6 Kinase protein were evaluated by Western blot. Representative blots from 3 independent experiments are shown.

RKO sh-control or sh-p32 cells were seeded on a 6-well plate and grown for 48 hours. After that time, the cells were starved for 16 hours with serum free media. Then the cells were stimulated with 10% FBS-DMEM for 1, 3 or 6 hours to evaluate the activation of Akt (**Figure 3A**), mTOR (**Figure 3B**) and p70-S6K (**Figure 3C**) proteins by Western blot. We found that mTOR activity and Akt activity were negatively and significantly affected in RKO -silenced cells compared with sh-control cells. As shown in **Figure 3A**, Akt phosphorylation diminished after 3 and 6 h post-incubation of cells with FBS-DMEM in sh-p32 cells in comparison with sh-control cells. The phosphorylated mTOR (Ser2448) and the Phosphorylation of its substrate p70-S6K also significantly decreased after 6 h of stimulation compared to sh-control cells (**Figures 3B, C**). As an additional test, the activation rate of Akt, mTOR and p70-S6K was evaluated in both RKO and SW480 cells at longer times of stimulation with 10% FBS (48 hours). As it can be observed in **Figure 3D**, in both cell types the rate of activation by phosphorylation of Akt, mTOR and p70-S6K was lower in the p32-depleted cells compared to the control cells. These results clearly indicated that knockdown of p32 inhibits mTOR activity in both RKO and SW480 colon cancer cells and are consistent with the role of p32 as an important promoter of malignant phenotype.

### Knockdown of p32 Affects the Migratory Capacity of RKO and SW480 Cells

**Figure 4:**
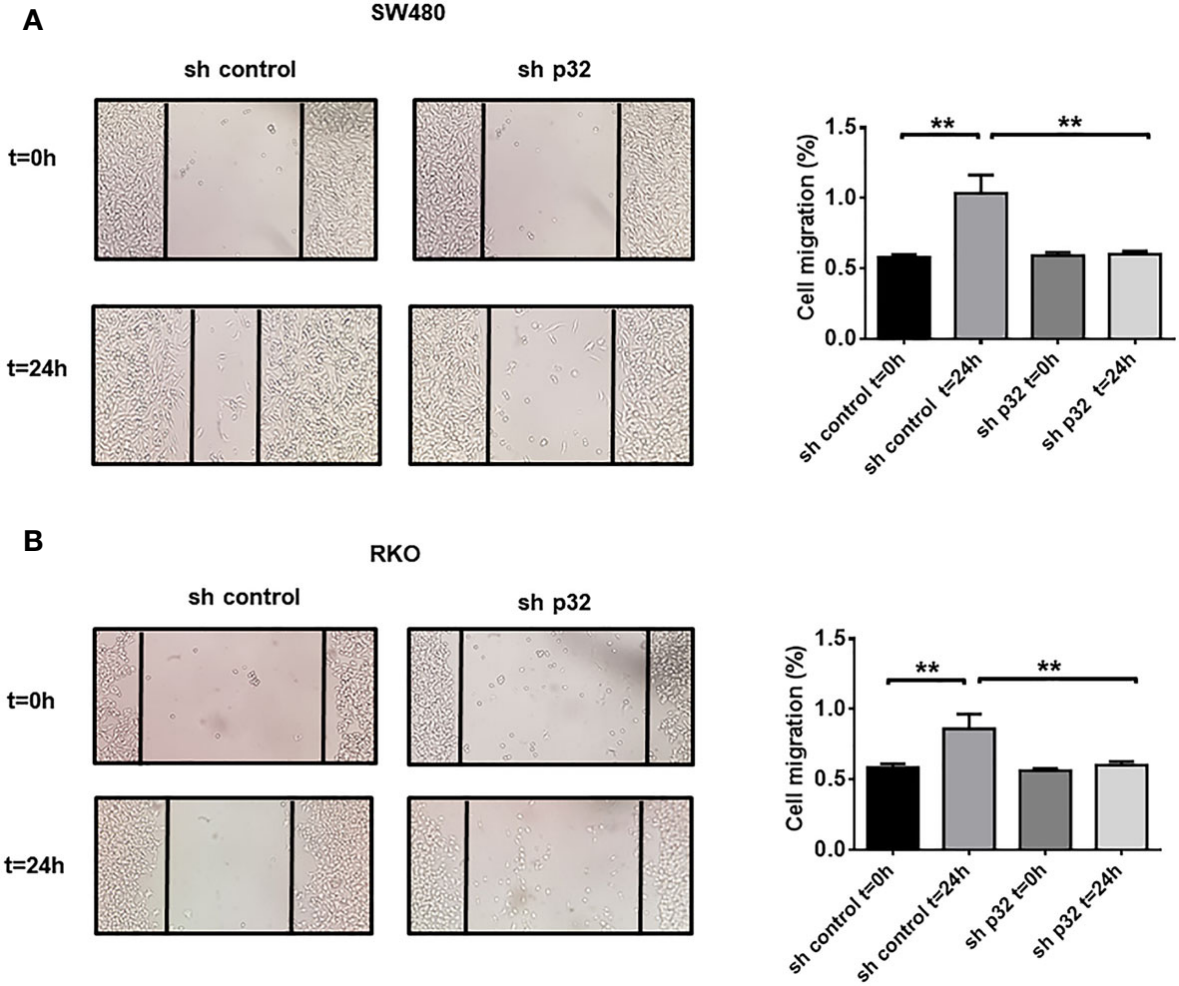
Knockdown of p32 affects the migratory capacity of SW480 and RKO cells. Wound healing assay for SW480-sh control and SW480 sh-p32 **(A)** or RKO sh-control and RKO sh-p32 **(B)** was performed as described under the *Materials and Methods* section. Representative images from 3 independent experiments are shown. The data is presented as the mean values ± SEM from at least three independent experiments, **p < 0.01.

Because Caveolin Lipid Rafts seem to be the preferred docking site for specific proteins involved in focal adhesion and cancer metastasis, we also evaluate the participation of p32 in migration processes in colon cancer cells. To this end, wound healing assays were carried out to compare the migratory ability of RKO and SW480 sh-control with that of p32-silenced cells. Knockdown of p32 induced a significant decrease in migratory capacity 24 hours after wound opening and in the absence of fetal bovine serum in the SW480 cell line (**Figure 4A**). Similar results were obtained for the RKO cell line, although with less significant effect than in SW480 cells (**Figure 4B**). These results clearly showed that p32 enhances the migratory capacity of RKO and SW480 colon cancer cells.

### Knockdown of p32 Sensitizes RKO and SW480 Cells to Cell Death Induced by Oxidative Stress and Chemotherapeutic Agents but Not to Death Induced by Nutritional Stress

**Figure 5:**
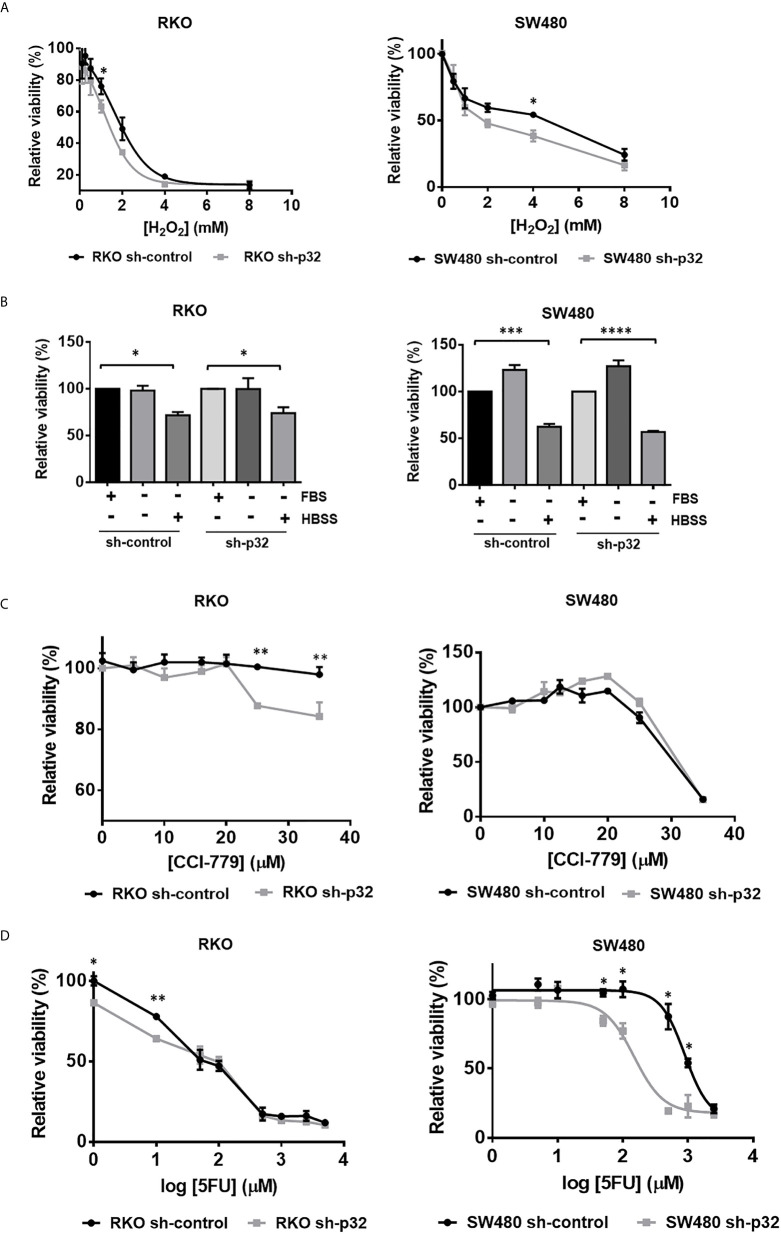
Knockdown of p32 sensitizes RKO and SW480 cells to cell death induced by oxidative stress and chemotherapeutic agents but not to death induced by nutritional stress. **(A)** RKO sh-control and RKO sh-p32 cells were seeded at 1.8 x10^4^ cells per well in 96-well-plate and cultured for 24 hours. The cells were then treated with H_2_O_2_ in concentrations of 8, 4, 2, 1, 0.5, 0.25, 0.125, 0 mM. After 12 h, the MTT assay was performed as described under “Materials and methods” section. The data was fitted to a dose-response inhibition curve and the EC50 was estimated for each condition. The estimated EC50s for RKO sh-control = 1.927 mM and for RKO sh-p32 = 1.051 mM. Each point on the curve represents the mean ± SEM of three independent experiments. *p <0.05. SW480 sh-control and SW480 sh-p32 cells were seeded at 2 x10^4^ cells per well in 96-well-plate and cultured for 48 hours. The cells were then treated with H_2_O_2_ in concentrations of 8, 4, 2, 1, 0.5 and 0 mM. After 16 h, the MTT assay was performed as described under *Materials and Methods* section. Each point on the curve represents the mean ± SEM of three independent experiments. *p < 0.05 **(B)** RKO sh-control and RKO sh-p32 cells were seeded at 1.8 x10^4^ cells per well in 96-well-plate and cultured for 24 hours. The cells were then treated with serum free DMEM (starving), Hanks’ balanced salt solution (HBSS) or complete DMEM (with FBS) as a control. After 12 h, the MTT assay was performed as described under *Materials and Methods* section. The data are presented as the mean values ± SEM from at least three independent experiments, *p < 0.05. SW480 sh-control and SW480 sh-p32 cells were seeded at 2 x10^4^ cells per well in 96-well-plate and cultured for 48 hours. The cells were then treated with serum free DMEM (starving), Hanks’ balanced salt solution (HBSS) or complete DMEM (with FBS) as a control. After 48 h, the MTT assay was performed as described under *Materials and methods* section. The data are presented as the mean values ± SEM from at least three independent experiments, ***p < 0.005, ****p < 0.001. **(C)** RKO sh-control and RKO sh-p32 cells were seeded at 1.8 x10^4^ cells per well in 96-well-plate and cultured for 24 hours. The cells were then treated with CCI-779 in concentrations of 0, 5, 10, 16, 20, 25, 35 μM. After 24 h, the MTT assay was performed as described under *Materials and Methods* section. Each point on the curve represents the mean ± SEM of three independent experiments. **p < 0.01. SW480 sh-control and SW480 sh-p32 cells were seeded at 2 x10^4^ cells per well in 96-well-plate and cultured for 48 hours. The cells were then treated with CCI-779 in concentrations of 0, 5, 10, 12.5, 16, 20, 25, 35 μM. After 24 h, the MTT assay was performed as described under *Materials and Methods* section. Each point on the curve is shown as the mean ± SEM of three independent experiments. **(D)** RKO sh-control and RKO sh-p32 cells were seeded at 1.8 x10^4^ cells per well in 96-well-plate and cultured for 24 hours. The cells were then treated with 5-fluoruracile (5FU) at concentrations of 0, 1, 10, 50, 100, 500, 1000, 2500, 5000 μM. After 72 h, the MTT assay was performed as described under *Materials and Methods* section. Each point on the curve is shown as the mean ± SEM of three independent experiments, *p < 0.05, **p < 0.01. SW480 sh-control and SW480 sh-p32 cells were seeded at 2 x10^4^ cells per well in 96-well-plate and cultured for 48 hours. The cells were then treated with 5-fluoruracile (5FU) at concentrations of 0, 1, 10, 50, 100, 500, 1000, 2500 μM. After 72 h, the MTT assay was performed as described under *Materials and Methods* section. The data was fitted to a dose-response inhibition curve and the EC50 was estimated for each condition. The estimated EC50s are: EC50 SW480 sh-control = 897.5 µM and EC50 SW480 sh-p32 = 153.5 µM. Each point on the curve is shown as the mean ± SEM of three independent experiments, *p < 0.05.

To further investigate the role of p32 in colon cancer cell malignancy, we next examined the effects of p32 on the resistance to cell death ability of RKO and SW480 colon cancer cell lines facing different types of stresses. First, we evaluated the cell viability measured by MTT of RKO or SW480 sh-control and sh-p32 cells treated in the absence or the presence of increasing concentrations of H_2_O_2_. As shown in **Figure 5A**, the viability analysis showed that for both conditions, there was a dose-dependent decrease of viability in each cell line where at extreme concentrations, both curves overlapped. However, for intermediate values of the curves, there were statistically significant differences in the degree of viability observed. Consistent with this, the IC50 values obtained in RKO cells were 1.927 mM for sh-control and 1.051 mM for sh-p32, while for SW480 cells were 4.6 mM for sh-control and 1.9 mM for sh-p32. This means that approximately twice the concentration of H_2_O_2_ is needed to induce the death of 50% of either RKO or SW480 sh-control cells compared to what is necessary to induce the death of 50% of RKO or SW480 sh-p32 cells (**Figure 5A**). Thus, these results indicate that p32 silencing sensitized both RKO and SW480 malignant cells to death induced by oxidative stress.

We further explored if the knockdown of p32 also sensitizes colon cancer cells to cell death induced by nutritional stress or by chemotherapeutic agents. To assess the effect of nutritional stress, RKO or SW480 sh-control and sh-p32 cells were cultured for 12 h or 48h in serum-free DMEM (starving) culture medium, or in Hanks’ balanced salt solution (HBSS), in comparison with complete DMEM culture medium as a control. Cell survival was evaluated using the MTT technique. As it can be observed in **Figure 5B**, the starving condition for 12 or 48 hours did not generate decrease in cell survival with respect to the control neither in RKO or SW480 sh-control cells nor in the RKO or SW480 sh-p32 cells. On the other hand, culturing cells in HBSS for 12 hours generated a significant decrease in viability in both RKO or SW480 sh-control and RKO or SW480 sh-p32 with respect to the control cells cultivated in complete DMEM. However, no significant differences were found between the viability of RKO or SW480 sh-control and sh-p32 treated with HBSS (**Figure 5B**). Similar results were obtained when the experiment was carried out at longer times (data not shown). Therefore, these results indicated that knockdown of p32 does not generate sensitivity to cell death induced by nutritional stress in colon cancer cells.

Next, to investigate if knockdown of p32 sensitizes malignant cells to death induced by the chemotherapeutic agents CCI-779 (Temsirolimus, an inhibitor of mTOR) or 5-FU (inhibitor of thymidine biosynthesis), we treated RKO or SW480 sh-control and RKO or SW480 sh-p32 cells with increasing concentrations of CCI-779 (**Figure 5C**) or 5-FU (**Figure 5D**) for 24 h or 72 h, respectively. After that time, we tested the cell viability by the MTT technique. Interestingly, as reported previously (36), the resistance displayed by RKO colon cancer cells against 5-FU or CCI-779 treatment was the opposite of that observed for SW480 cells (**Figures 5C, D**), since RKO sh-control cells displayed resistance to CCI-779 treatment but were 5-FU sensitive, while SW480 cells were sensitive to CCI-779 but displayed resistance to 5-FU, since doses lower than 100 μM did not significantly inhibit cell viability. Remarkably, as it can be observed in **Figures 5C, D**, the knockdown of p32 expression reverted the resistance to these agents, since sensitized RKO cells to death induced by CCI-779 at doses over 20 μM and sensitized also SW480 cells to 5-FU treatment compared with sh-control cells: the IC50 found for SW480 sh-control cells was 897.5 µM, about 6 times higher than the estimated for sh-p32 cells with the same treatment (153.5 µM).

To assess the effect of oxidative stress or cytotoxic stress (induced by CCI-779 or 5-FU) on apoptosis induction, the cells were examined by Western blotting to detect the levels of cleaved caspase-3 or cleaved PARP. **Supplementary Figure 1** shows how, as expected, oxidative stress or drug treatment of RKO sh-control cells increased apoptotic cell death visualized as an increase in the levels of cleaved caspase-3 or cleaved PARP. But strikingly, it also shows that under the same stressful conditions, the apoptosis rate visualized as cleaved caspase-3 or cleaved PARP was higher in p32-depleted cells compared to sh-control cells (**Supplementary Figure 1**). Consistent with this, the micrographs shown in **Supplementary Figure 1** demonstrate a higher sensitization to apoptotic death of p32-depleted cells than found in control cells. Taken together, the data indicate that p32 promotes resistance to cell death induced by oxidative stress (H_2_O_2_) and chemotherapeutic agents (CCI-779 or 5-FU), but not to cell death induced by nutritional stress.

### Knockdown of p32 Affects the Clonogenic and Tumorigenic Ability of Both RKO and SW480 Colon Cancer Cells

**Figure 6:**
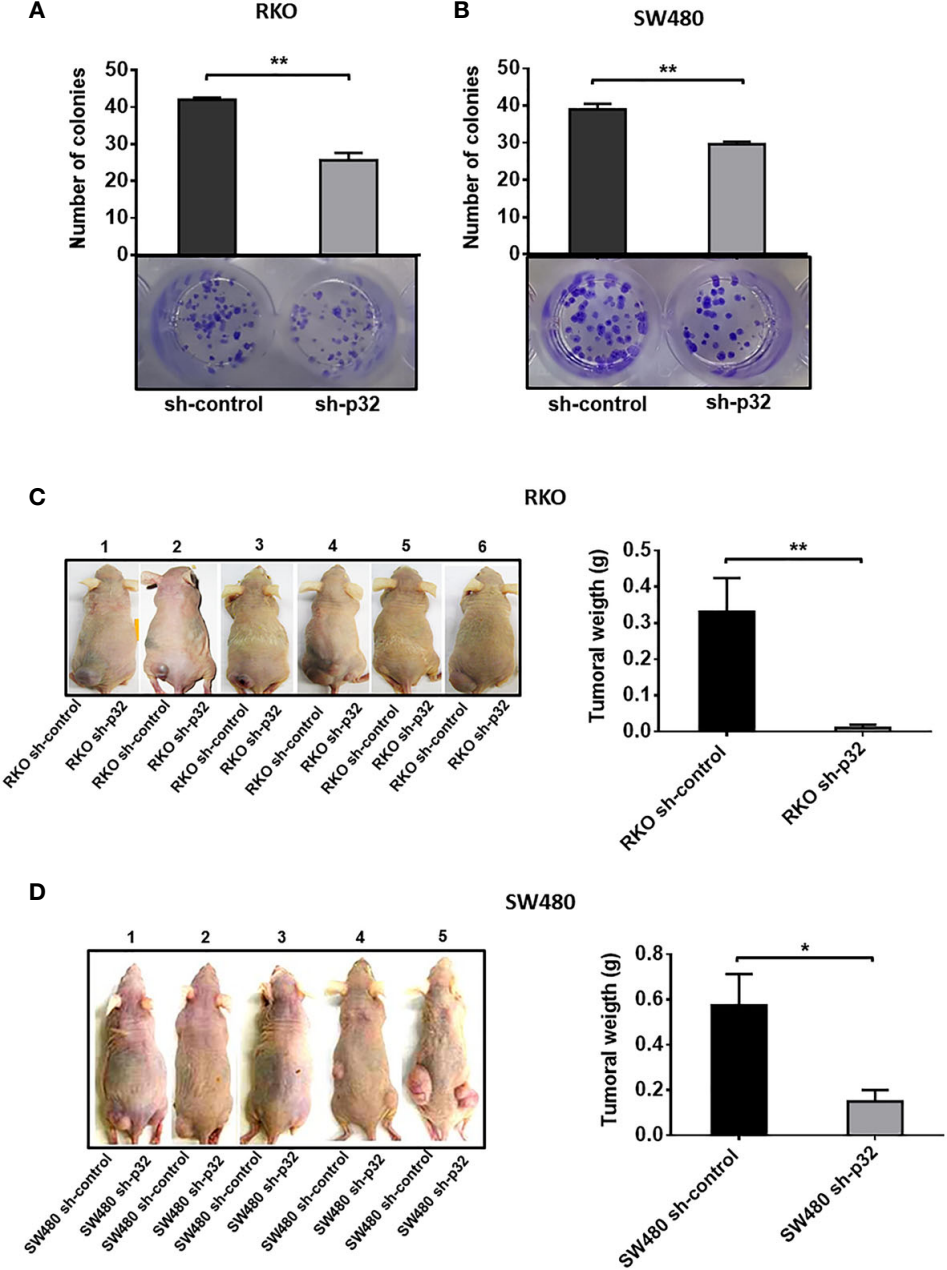
Knockdown of p32 affects the clonogenic and tumorigenic capacity of RKO and SW480 cells. Colony formation assays for RKO sh-control and RKO-sh p32 cells **(A)** or SW480 sh-control cells and SW480 sh-p32 cells **(B)** were performed as described under the *Material and Methods* section. Representative photographs from 3 independent experiments are shown. The data is presented as the mean values ± SEM from three independent experiments, **p < 0.01. **(C)** The tumorigenic capacity of RKO sh-control and RKO sh-p32 cells was evaluated in 6 nude mice as described in the *Materials and Methods* section. Photographs taken of the experimental animals with tumors just after euthanasia are shown. The graph represents the mean ± SEM of the weights of tumors formed by the RKO sh-control or RKO sh-p32 cells on the 6 experimental animals, **p < 0.01. **(D)** The tumorigenic capacity of SW480 sh-control and SW480 sh-p32 cells was evaluated in 5 nude mice as described in the *Materials and Methods* section. Photographs taken of the experimental animals with tumors just after euthanasia are shown. The graph represents the mean ± SEM of the weights of tumors formed by the SW480 sh-control or SW480 sh-p32 cells on the 5 experimental animals, *p < 0.05.

We investigated if p32 knockdown affects the clonogenic capacity of colon cancer cells. To achieve this, we performed a colony formation assay. The photographs and their corresponding bar graphs are shown in **Figures 6A, B**. The results demonstrated that RKO or SW480 sh-control cells displayed higher clonogenic capacity than RKO or SW480 sh-p32 since in each case sh-control cells were able to form almost twice the number of colonies formed by p32-depleted cells (**Figures 6A, B**). Finally, to prove *in vivo* whether p32 knockdown affects the tumorigenic capacity of colon cancer cells, we made use of a xenograft model in nude mice. Six immunocompromised mice were injected in the left flank subcutaneously (s.c.) with 1 X 10^6^ RKO sh-control cells and in the right flank with 1 X 10^6^ RKO p32-silenced cells. The same protocol was followed injecting mice with SW480 sh-control or sh-p32 cells. After three weeks, the mice were euthanized, and the tumors formed in each case were weighed. It was found that control colon cancer cells (expressing p32) were able to form tumors with a significantly higher weight than tumors formed by cells with depleted expression of p32 (**Figures 6C, D**). Therefore, these experiments clearly showed that the knockdown of p32 protein negatively affects the tumorigenic capacity of colon cancer cells. Taken together, our results indicate that p32 promotes the clonogenic and tumorigenic capacity of colon cancer cells. In addition, the data shown here indicate that p32 plays a crucial role in promoting malignant phenotype in colon cancer cells.

## Discussion

In this study we demonstrated that over-expression of p32 promotes cell migration, resistance to cell death induced by oxidative stress and chemotherapeutic agents, clonogenic capacity and *in vivo* tumorigenesis of colorectal cancer cells. Interestingly, these functions are closely related to the intracellular localization of p32 in colon cancer cells.

Although p32 is a protein that is located primarily at the mitochondria, it has also been found located at the plasma membrane and at the nucleus by multiple authors (13, 14, 37). The PI3K/AKT pathway and Ras/ERK signaling pathways are frequently dysregulated in colon cancer. In this study, we employed SW480 cells as the prototype of KRAS-driven colon cancer cells and RKO as the prototype of BRAF-driven colon cancer cells. Remarkably, we observed that the expression blockade of p32 negatively affects the activation of both Akt and mTORC proteins. This means that p32 enhances the activation of these oncogenic signaling pathways converging in mTORC, and thus favors cell survival, proliferation and growth. The molecular mechanism by which p32 may enhance mTOR-coupled signaling pathways can be explained by its significant presence in caveolin lipid rafts (CLR), since Arielly SS. et al. (34) have reported that p32 is the most abundant protein present in CLRs of colon cancer cells. Consistent with this, other groups have reported that in pancreatic and lung cancer cells, growth factors induce the translocation of p32 to membrane lipid rafts where its interaction with CD44 favors the autophosphorylation and activation of Receptor Tyrosine Kinases (RTKs) (27, 29). In addition, the location of p32 at the membrane CLRs would also explain its participation in cell migration. In this regard, it is well known that CLRs are the preferred docking sites for specific proteins involved in focal adhesion and cancer metastasis (27, 35, 38). In agreement with this, here we found that p32 expression depletion induced a significant decrease in migratory ability of colon cancer cells.

Regarding the functions of p32 located at the cell nucleus, we found greater nuclear localization of the protein in malignant cells than in non-malignant ones as it can be observed in **Figure 1**. This may have important functional implications for the protein in the context of cancer. At this location, it has been assigned to perform functions such as splicing regulation (23) and transcriptional activity modulation of various transcription factors such as FOXC1 (39) and TFIIB (40). Interestingly, recent work has shown that the interaction of p32 with the important tumor suppressor p53 at the nucleus inhibits p53 tetramerization and transcription of its target genes (14). Therefore, it is not surprising that as a consequence of the knockdown of p32 in RKO malignant cells (expressing wild type p53), the transcription levels of genes closely linked to malignant phenotypes are also altered (**Supplementary Tables 1** and **2**, **Table 1**, **Figure 2**). It is possible that p32 indirectly regulates the expression of these genes in colorectal cancer cells by modulating the activity of different transcription factors such as p53 at the nucleus. In addition, p32 may affect the activity of proteins involved in important signaling pathways such as Protein Kinase C isoforms (41, 42). By modulating the activity of these signaling proteins, p32 could also modify the expression of genes associated with the malignant phenotype.

Among the most important genes whose expression was found modified by the knockdown of p32 in RKO cells are HAS-2 (hyaluronic acid synthase-2) which was downregulated, and PDCD4, which was upregulated (**Table 1**, **Figure 2**). Interestingly, HAS-2 has recently been found as an important promoter of malignant phenotype in colorectal cancer cells through enhancing metastatic capacity, resistance to cell death, epithelial-mesenchymal transition, among other functions (43). Our data suggest that p32 induces increases in the expression levels of HAS-2 and CD44. HAS-2 is an enzyme whose fundamental function is the synthesis of hyaluronic acid (HA) from the cell surface. Hyaluronic acid has been closely linked to the increased migratory and metastatic capacity of cancer cells. In addition, CD44 is a molecule whose main ligand is hyaluronic acid and promotes migration and metastasis in different cancer types (33). It is possible that the pro-migration effect exerted by p32 is in part supported by its ability to increase the levels of each component of the HAS-2/HA/CD44 molecular system. The fact that p32 promotes the migratory capacity of colorectal cancer cells strongly suggests that this protein may play a pro-metastatic role in this type of malignancy. On the other hand, PDCD4 is an important tumor suppressor gene, which is found in low levels or absent in colon cancer cells. Decreased PDCD4 levels have been associated with increased proliferation, migration, and metastasis and decreased apoptosis in colorectal malignancies (44). Our data suggest that p32 participates in the down-regulation of PDCD4 in colon cancer cells since when p32 expression is blocked in RKO cells, an increase in PDCD4 levels is generated (**Table 1**). Therefore, malignancy driver functions attributed in our work to p32 in colorectal cancer cells could be explained at least partly by its ability to significantly modify the transcription levels of *HAS-2* and *PDCD4* genes, and to its ability to activate the PI3K/Akt/mTOR signaling pathway.

It was also found that knockdown of p32 sensitizes colon cancer cells to apoptotic cell death induced by oxidative stress and chemotherapeutic agents such as CCI-779 (Temsirolimus) and 5-FU (**Figure 4** and **Supplementary Figure 1**). The anti-apoptotic role of p32 can be the result of its participation as a positive modulator of Akt/mTORC signaling pathway. In addition, the death-protective functions that p32 performs are possibly enhanced in colon cancer cells by its role as a regulator of the expression of proteins associated with a malignant phenotype. HAS-2, a protein whose levels are upregulated by p32, has recently been implicated in resistance to cell death induced by radiation and oxaliplatin (43). On the other hand, PDCD4, which is downregulated by p32, is an important promoter of cellular apoptosis induced by different factors (44). Interestingly, inhibition of PDCD4 by micro-RNA mir21 has been involved in 5-FU drug resistance in RKO cells (45). Thus, it is highly probable that p32 cytoprotective effects against 5-FU are at least in part mediated by the ability of p32 to inhibit PDCD4 expression. Surprisingly, knockdown of p32 did not sensitize colon cancer cells to death induced by nutritional stress (**Figure 5B**). A possible explanation of this could be that acute nutritional stress produced by incubating cells in HBSS at long periods of time (higher than 8 hours) mainly induces autophagy cell death (autosis), and that p32 regulates the death process associated with mitochondria such as apoptosis, but does not participate in autosis cell death. Additional studies will be necessary to understand further the mechanisms underlying the cytoprotective role of p32.

Finally, we demonstrated here that p32 enhances the clonogenic and tumorigenic capacity *in vivo* of colon malignant cells (**Figure 6**). The pro-malignancy effects observed of p32 are even more marked *in vivo* than *in vitro* experiments, which suggests that in addition to regulation of genes associated with the malignant phenotype, increased migration, resistance to cell death, and clonogenic capacity, other factors could be enhancing the *in vivo* tumorigenic role of p32.

Taken together, our data demonstrate that p32 plays an important role in promoting malignant phenotype in CRC cells, strongly suggesting that p32 can be used as a therapeutic target in CRC treatment. The development of neutralizing antibodies or specific inhibitors of p32 could be a good strategy that in fact, has already begun in other cancer types (46, 47). Therefore, the task of testing these methods and developing new strategies to block the pro-malignant functions of p32 is promising in colorectal cancer treatment.

## Data Availability Statement

The datasets presented in this study can be found in online repositories. The names of the repository/repositories and accession number(s) can be found in the article/**Supplementary Material**.

## Ethics Statement

The animal study was reviewed and approved by the Faculty of Medicine Ethical Committee at the Universidad Nacional Autónoma de México (in accordance to the Mexican Official Norm NOM-062-ZOO-1999).

## Author Contributions 

Conceptualization, MR-F and CE-A. Formal analysis, CE-A, VM, JM-Z, SA-G, and MR-F. Funding acquisition, MR-F. Investigation, CE-A, VM, and MC-P, and MR-F. Methodology, CE-A, MC-P, SA-G, HG-A, APM-L, VM, and JM-Z. Project administration, MR-F. Resources, VM and MR-F. Software, CE-A, SA-G, VM, and JM-Z. Supervision, MR-F. Validation, CE-A, SA-G, MC-P, and MR-F. Visualization, CE-A and HG-A. Writing—original draft, CE-A and MR-F. All authors contributed to the article and approved the submitted version.

## Funding

This research was supported by grants from Universidad Nacional Autónoma de México (DGAPA-UNAM IV200220 and IN229420) and from CONACYT (FOSSIS 2017-289600).

## Conflict of Interest

The authors declare that the research was conducted in the absence of any commercial or financial relationships that could be construed as a potential conflict of interest.

## Supplementary Material

Supplementary Figure 1 -Knockdown of p32 sensitizes RKO cells to apoptotic cell death induced by oxidative stress and chemotherapeutic agents CCI-779 and 5FU **(A)** Representative micrographs of RKO sh-control and RKO sh-p32 cells treated with 1 mM H_2_O_2_ for 12 h. Scale bar, 50 μm. **(B)** Western blot analysis for the activation of Caspase-3 by proteolytic cleavage in RKO sh-control and RKO sh-p32 cells treated with 1 mM H_2_O_2_ for 12 h. **(C)** Representative micrographs of RKO sh-control and RKO sh-p32 cells treated with 35 μM CCI-779 for 24 h. Scale bar, 50 μm. **(D)** Western blot analysis for the activation of PARP by proteolytic cleavage in RKO sh-control and RKO sh-p32 cells treated with 25 μM CCI-779 for 12 or 24 h. **(E)** Representative micrographs of RKO sh-control and RKO sh-p32 cells treated with 1 μM 5FU for 72 h. Scale bar, 50 μm. **(F)** Western blot analysis for the activation of Caspase-3 and PARP by proteolytic cleavage in RKO sh-control and RKO sh-p32 cells treated with 10 μM 5FU for 48 or 72 h.
