# Supplementary figures and images for: Overexpression of Multifunctional Protein p32 Promotes a Malignant Phenotype in Colorectal Cancer Cells
Source: Front Oncol. 2021 May 31;11:642940. doi: 10.3389/fonc.2021.642940 (PMC8201776; doi:10.3389/fonc.2021.642940)

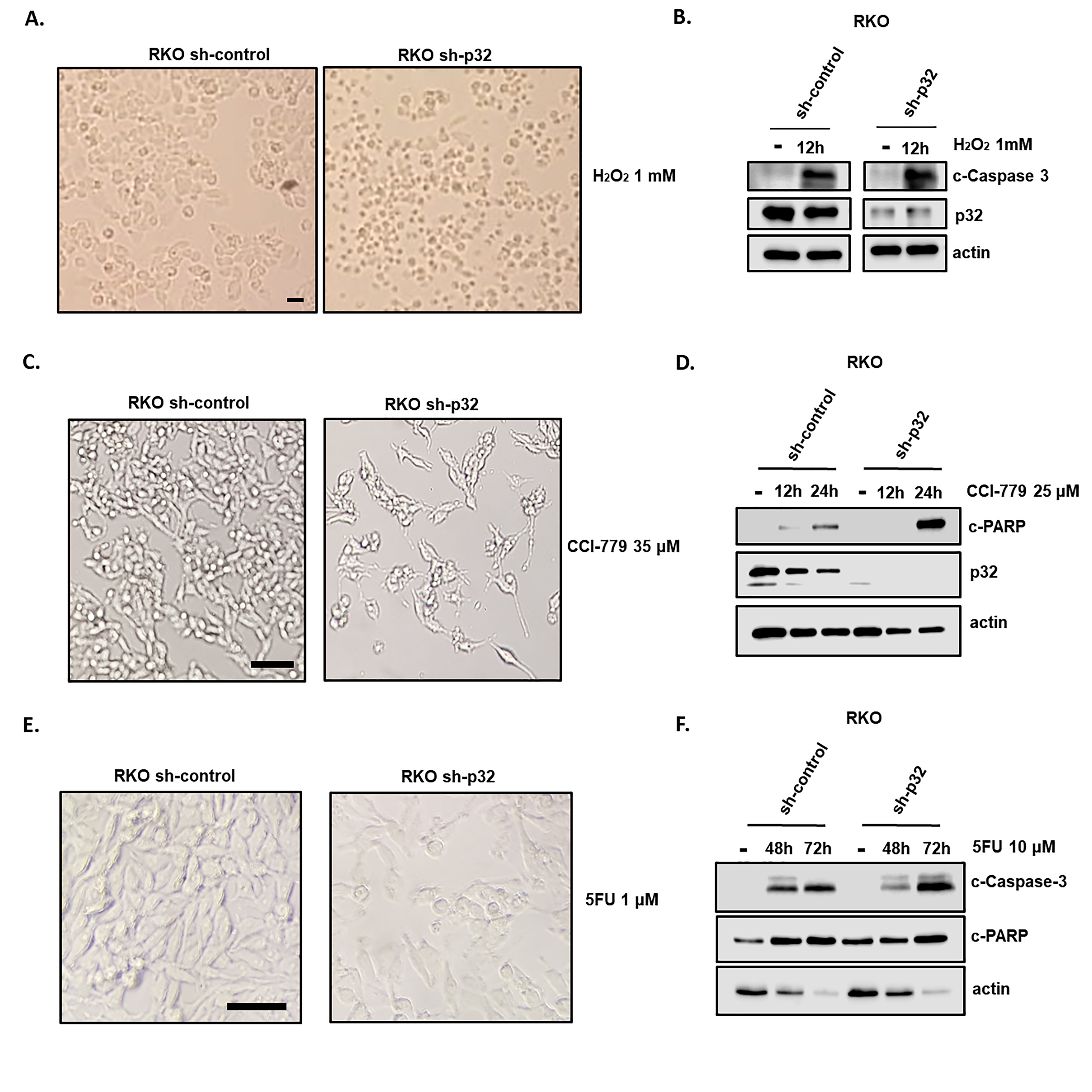

Supplement: Supplementary Figure 1 — -Knockdown of p32 sensitizes RKO cells to apoptotic cell death induced by oxidative stress and chemotherapeutic agents CCI-779 and 5FU (A) Representative micrographs of RKO sh-control and RKO sh-p32 cells treated with 1 mM H2O2 for 12 h. Scale bar, 50 μm. (B) Western blot analysis for the activation of Caspase-3 by proteolytic cleavage in RKO sh-control and RKO sh-p32 cells treated with 1 mM H2O2 for 12 h. (C) Representative micrographs of RKO sh-control and RKO sh-p32 cells treated with 35 μM CCI-779 for 24 h. Scale bar, 50 μm. (D) Western blot analysis for the activation of PARP by proteolytic cleavage in RKO sh-control and RKO sh-p32 cells treated with 25 μM CCI-779 for 12 or 24 h. (E) Representative micrographs of RKO sh-control and RKO sh-p32 cells treated with 1 μM 5FU for 72 h. Scale bar, 50 μm. (F) Western blot analysis for the activation of Caspase-3 and PARP by proteolytic cleavage in RKO sh-control and RKO sh-p32 cells treated with 10 μM 5FU for 48 or 72 h. [file Image_1.tif]
